# Supplementary material for: easyEWAS: a flexible and user-friendly R package for epigenome-wide association study
Source: Bioinform Adv. 2025 Feb 13;5(1):vbaf026. doi: 10.1093/bioadv/vbaf026 (PMC11878637; doi:10.1093/bioadv/vbaf026)
Supplement: vbaf026_Supplementary_Data [file vbaf026_supplementary_data.zip › Supplement Figures.pdf]

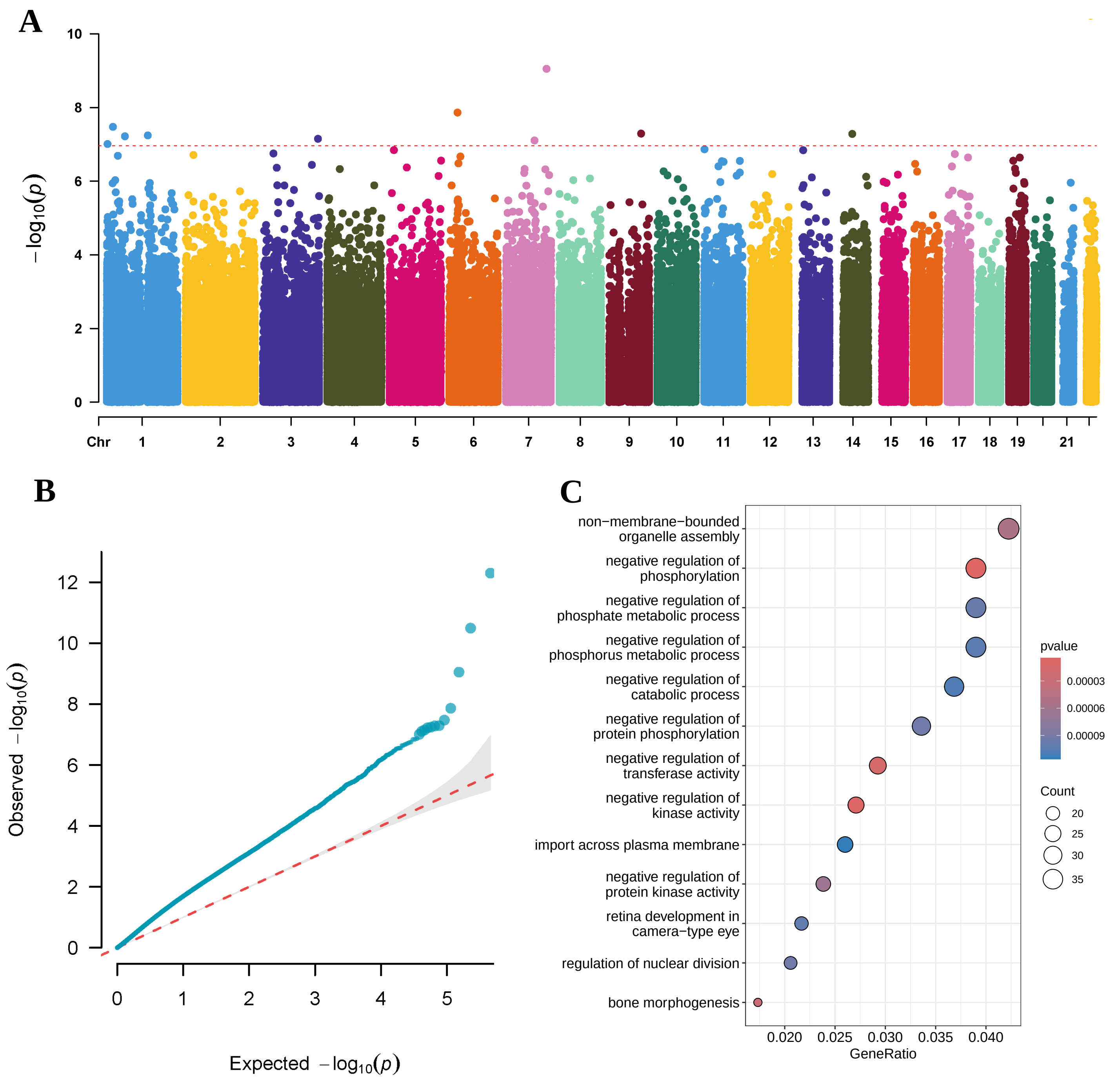

Figure S1 The results obtained from EWAS analyses in asthmatic patients using *easyEWAS*. **(A)** Rectangular Manhattan plot showing p-values for epigenome-wide association studies comparing DNA methylation patterns between asthma patients and healthy controls. The red dotted line indicates the p-value threshold of the Bonferroni correction method. Abbreviations: Chr, chromosome. **(B)** EWAS quantile-quantile plot showing inflation factor with 95% confidence intervals. **(C)** Bubble plot of GO enrichment analysis of differentially methylated genes.

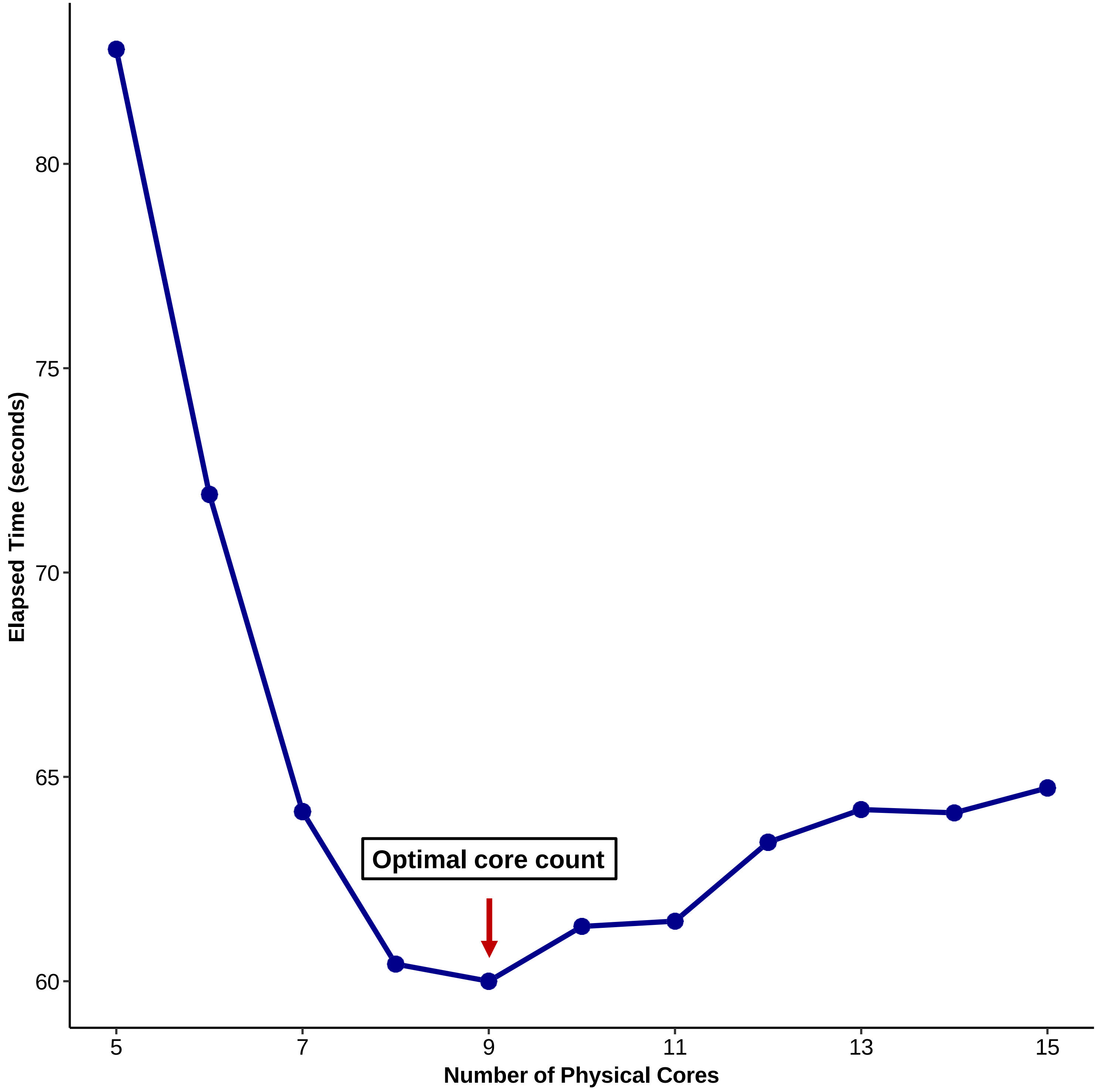

Figure S2 Benchmarking analysis of core usage for EWAS runtime optimization.
